# Angiotensin-converting-enzyme inhibitors (ACE inhibitors) and angiotensin II receptor blocker (ARB) use in COVID-19 prevention or treatment: A paradox

**DOI:** 10.1017/ice.2020.195

**Published:** 2020-05-04

**Authors:** Shaghayegh Haghjooy Javanmard, Kiyan Heshmat-Ghahdarijani, Golnaz Vaseghi

**Affiliations:** 1 Applied Physiology Research Center, Cardiovascular Research Institute, Isfahan University of Medical Sciences, Isfahan, Iran; 2 Heart Failure Research Center, Cardiovascular Research Institute, Isfahan University of Medical Sciences, Isfahan, Iran; 3 Isfahan Cardiovascular Research Center, Cardiovascular Research Institute, Isfahan University of Medical Sciences, Isfahan, Iran

*To the Editor—*Coronavirus disease 2019 (COVID-19), which affects type II alveolar cells of the human lung, is caused by severe acute respiratory syndrome coronavirus 2 (SARS-CoV-2).^1^ This virus was identified in December 2019 in Wuhan, China, for the first time,^2^ and has spread all over the world, leading to a global pandemic.^3^

Renin-angiotensin system (RAS) signaling and angiotensin-converting enzyme 2 (ACE2) have been implicated in the pathogenesis of COVID-19.^4^ The virus binds to its target cells through angiotensin-converting enzyme 2 (ACE2), which is found in the type II alveolar cells of the lungs. Furthermore, ACE2 receptors are expressed in many extrapulmonary tissues such as heart, kidney, testis, endothelia, and the gastrointestinal tract.^5,6^ ACE2-expressing alveolar cells are also involved in the viral genome replication process.^7^

ACE2 degrades angiotensin (ANG) I to angiotensin (1–9), which is a ligand for angiotensin II receptor type 2 (AT_2_). ANG (1–9) has regenerative and anti-inflammatory effects through its binding to the AT_2_ receptor.^8^ Moreover, ACE2 converts angiotensin II to angiotensin (1–7).^9^ ANG (1–7), through binding to the Mas receptor (MasR), mediates anti-inflammatory and vasodilatory effects and reduces reactive oxygen species (ROS). Thus, it counteracts the vasoconstriction and proinflammatory effects of ANG II.^10,11^ In addition, ANG (1–7) may prevent lung injury because of its vasodilator effect.^12^

Importantly, SARS-CoV infections and SARS spike protein downregulate ACE2 expression.^13^ Furthermore, blocking the RAS pathway deteriorated acute lung injury induced by the injection of SARS-CoV spike protein in mice. Thus, in contrast to most other coronaviruses, SARS-CoV may have become highly lethal because the virus dysregulates a lung protective pathway.^14^

Animal studies have shown that ACE2 protects murine lungs from acute lung injury as well as reconciles SARS spike protein lung injury, suggesting a dual role of ACE2 in both SARS infections and protection from ARDS.^12^ The effect of ACE inhibitors (ACE-I) and angiotensin II type-I receptor blockers (ARBs) in the treatment and prevention of COVID-19 is not well recognized.

ACE-I and ARBs have been shown to upregulate the expression of ACE-2 or to prevent the loss of ACE2 in the heart; they may have a similar effect in lung tissue.^15,16^ Thus, an ACE-I and ARB blocker prescription in COVID-19 patient may make the patient vulnerable due to additional virus entrance and replication in type II alveolar cells.

Hypertension, diabetes, and coronary heart disease were the most common comorbidities associated with death from COVID-19 in Wuhan patients,^17^ which leads us to 2 paradoxical hypotheses:

1. The expression of ACE2 in hypertensive patients and patients with type 1 or type 2 diabetes, who are treated with ACE inhibitors ARBs, is increased.^18^ This upregulation may make these patients more vulnerable.
2. On the other hand, diabetes and hypertension are associated with decreased baseline levels of ACE2 expression.^18^ Therefore, SARS-CoV-2 binding to ACE2 may decrease residual ACE2 activity and lead to a predominance of ANG II through AT1 receptor signaling. In this case, ANG II causes pulmonary vasoconstriction and inflammatory and oxidative organ damage, ultimately progressing toward ARDS.^19^

Although some beneficial experimental evidence has emerged regarding ACEI or ARBs, and their use is well tolerated, inexpensive, and widespread; the potential therapeutic effects of these drugs in ARDS caused by SARS-CoV-2 is doubtful.

However, multiple regulatory associations have recommended that hypertensive COVID-19 patients do not stop taking their previosuly prescribed ACE inhibitors or ARBs.^20^ The evidence offered here precedes any clinical trials, and the paradoxical role of the aforementioned drugs should be solved in preclinical and epidemiological studies.
